# Is breast cancer prognosis inherited?

**DOI:** 10.1186/bcr1737

**Published:** 2007-06-28

**Authors:** Mikael Hartman, Linda Lindström, Paul W Dickman, Hans-Olov Adami, Per Hall, Kamila Czene

**Affiliations:** 1 Department of Medical Epidemiology and Biostatistics, Karolinska Institutet, P. O. Box 281, 171 77 Stockholm, Sweden; 2 Stockholm Söder Hospital, Karolinska Institutet, Sjukhusbacken 10, 118 83 Stockholm, Sweden; 3 Department of Epidemiology, Harvard University, 677 Huntington Avenue, Boston, 02115 MA, USA

## Abstract

**Introduction:**

A genetic component is well established in the etiology of breast cancer. It is not well known, however, whether genetic traits also influence prognostic features of the malignant phenotype.

**Methods:**

We carried out a population-based cohort study in Sweden based on the nationwide Multi-Generation Register. Among all women with breast cancer diagnosed from 1961 to 2001, 2,787 mother-daughter pairs and 831 sister pairs with breast cancer were identified; we achieved complete follow-up and classified 5-year breast cancer-specific prognosis among proband (mother or oldest sister) into tertiles as poor, intermediary, or good. We used Kaplan-Meier estimates of survival proportions and Cox models to calculate relative risks of dying from breast cancer within 5 years depending on the proband's outcome.

**Results:**

The 5-year survival proportion among daughters whose mothers died within 5 years was 87% compared to 91% if the mother was alive (*p *= 0.03). Among sisters, the corresponding proportions were 70% and 88%, respectively (*p *= 0.001). After adjustment for potential confounders, daughters and sisters of a proband with poor prognosis had a 60% higher 5-year breast cancer mortality compared to those of a proband with good prognosis (hazard ratio [HR], 1.6; 95% confidence interval [CI], 1.2 to 2.2; *p *for trend 0.002). This association was slightly stronger among sisters (HR, 1.8; 95% CI, 1.0 to 3.4) than among daughters (HR, 1.6; 95% CI, 1.1 to 2.3).

**Conclusion:**

Breast cancer prognosis of a woman predicts the survival in her first-degree relatives with breast cancer. Our novel findings suggest that breast cancer prognosis might be inherited.

## Introduction

Breast cancer, the most common female malignancy, has an important genetic contribution estimated to 25% to 28% [1,2]. Mutations in high-penetrant genes such as *BRCA1 *(breast cancer 1, early onset) and *BRCA2 *account for only a small proportion of this hereditary component, suggesting an important but yet-to-be-detected role for low-penetrant single nucleotide polymorphisms. Overall, the prognosis of women with a family history of breast cancer has been reported as similar or worse compared to women without a family history [3-6]. A relatively poor outcome has been reported among *BRCA1*-positive women [6], but some controversy still remains [7]. There is also evidence that some non-genetic risk factors such as hormone replacement therapy (HRT) and obesity influence both the incidence [8-10] and the prognosis [11-13] of breast cancer.

We hypothesized that genetic traits may influence not only the risk of developing breast cancer but also prognostically important features of the malignant phenotype. To the best of our knowledge, this hypothesis has never been adequately investigated. To this end, we identified women with incident breast cancer and analyzed breast cancer prognosis among 2,787 mother-daughter pairs and 831 sister pairs, all of whom had breast cancer diagnosed in Sweden from 1961 to 2001. We achieved complete follow-up and unbiased information on family history through record linkages.

## Materials and methods

### Study cohort

The Multi-Generation Register includes all Swedish residents born after 1931, who were alive in 1960, and all those born thereafter. It contains links between children and parents through their national registration numbers, which are assigned to all residents of Sweden. From 1961 to 2001, the completeness of the Multi-Generation Register became progressively better, and from 1991 it is considered to be complete [14]. Therefore, among individuals who died before 1991, notification of their mothers in the Multi-Generation Register has some degree of incompleteness. Information on cancer was obtained from the nationwide Swedish Cancer Registry, established in 1958. For the period of our study (1961 to 2001), the cancer registry was estimated to be at least 98% complete [15]. For each notified cancer, the cancer registry records the national registration number, International Classification of Diseases code, date of diagnosis, and area of residence at diagnosis. Information on stage of disease and treatment is not included in the registry.

Further record linkages to the nationwide Cause of Death Register and the Total Population Register allowed complete follow-up with regard to vital status, date and underlying causes of death, as well as dates of emigration and immigration. Follow-up started in 1961 because the Cause of Death Register has a high reported accuracy (96%) [16] from that year onward. Finally, additional linkages were made to the censuses of 1960, 1970, 1980, and 1990, which contain information on the socioeconomic status of each Swedish citizen.

From a total population cohort of about 11 million individuals recorded in the Multi-Generation Register, we identified 36,554 female offspring who were born in Sweden since 1932 and who had a first primary invasive breast cancer diagnosed from 1961 to 2001. Subsequently, we identified all mothers and sisters of these women who were also born in Sweden and whose first primary invasive breast cancer was diagnosed during the same period. We excluded all women for whom the history of breast cancer was uncertain because they had immigrated to Sweden and all women with any primary malignant tumor other than a breast cancer prior to the first breast cancer. Our final dataset comprised a total of 2,787 mother-daughter pairs and 831 sister pairs with incident breast cancer.

Parity and age at first birth were extracted from information on offspring available in the Multi-Generation Register. Area of residence at diagnosis was obtained from the Cancer Registry and categorized into the six health care regions in Sweden. Socioeconomic status, estimated from the highest level of employment in the household reported in the censuses, was categorized into four groups: self-employed, blue-collar worker, and low-level and high-level white-collar worker.

### Statistical methods

All analyses are based on breast cancer-specific mortality among patients with an affected mother or sister (proband). We limited the outcome estimate to 5 years because it is a clinically accepted estimate of prognosis. The person-time at risk started at the date of first diagnosis of breast cancer and continued until emigration, end of follow-up (31 December 2001), or death, whichever came first. In the sister pair analysis, the older sister was chosen as the proband for reasons of better statistical power by an approximately equal number of deaths between the sister pairs. All of the data preparation and analysis were carried out using the SAS Statistical package, version 8.2 (SAS Institute Inc., Cary, NC, USA) [17].

Firstly, we conducted a univariable analysis crudely grouping the proband (mother or sister) into either dead due to breast cancer within 5 years of diagnosis or alive 5 years after diagnosis. Due to the end of follow-up of the register in December 2001, we restricted the date of diagnosis until 1996 to ensure that all probands had the possibility of 5-year survival. We used the Kaplan-Meier method to estimate survival proportions, looking at differences in cause-specific death depending on the proband's prognosis. To increase validity and to avoid confounding by period, we restricted follow-up for the daughters and sisters at risk to the years since 1991.

Secondly, our ultimate aim was to model the prognosis of the daughters and sisters as a function of the prognosis of the proband (mother or older sister, respectively). We first needed to classify the prognosis of the proband, and we did so based on the deviance residual from a multivariable proportional hazards (Cox) model fitted to the proband data adjusting for period and age of diagnosis. The deviance residual provides a measure of how the survival of the proband compares to other probands with the same age and year of diagnosis. Because the residual is calculated as observed minus expected mortality, values below, above, and around zero correspond to prognoses of better, worse, or as expected, respectively. The deviance residuals are more symmetrically distributed about zero than the unadjusted (crude) residuals. We defined the good prognosis group as the first tertile of the deviance residual distribution, the medium prognosis group as the second tertile, and the poor prognosis group as the third tertile. Finally, the association between the cause-specific hazard in the prognoses of daughters or sisters and probands was investigated employing a proportional hazards model adjusting for all available confounders such as age and calendar period of diagnosis, parity, age at first birth, socioeconomic factors, and area of residence at diagnosis.

Finally, due to limited statistical power to model the effect of age, we instead examined the concordance proportion in prognosis between mother-daughter pairs by using the modeled estimates of prognosis. We examined the relationship between the age of the mother at diagnosis and the concordance in prognosis (good, medium, or poor) and assessed the trend in relation to age.

## Results

### Univariable analysis

Table 1 presents patient characteristics of the breast cancer pairs. Mothers with breast cancer had a widely distributed range of age at diagnosis and period of diagnosis. In contrast, daughters and sisters with breast cancer were limited in age to less than 70 years and their tumors were diagnosed predominantly at the end of the study period. Information on parity and age at first birth is used to describe the risk of death in the daughters or younger sisters only.

**Table 1:** Characteristics of 2,787 mother-daughter pairs and 831 sister pairs with primary breast cancer in Sweden from 1961 to 2001

	Mother		Daughter		Older sister		Younger sister	
	Number	Percentage	Number	Percentage	Number	Percentage	Number	Percentage

Number	2,787		2,787		831		831	
Overall deaths	2,010	72.1	511	18.3	182	21.9	148	17.8
Cause-specific deaths	1,009	36.2	395	14.2	133	15.9	120	14.4
Age at diagnosis (years)								
<40	56	1.9	417	14.4	63	7.6	117	14.1
40–49	305	10.6	1,148	39.8	264	31.8	346	41.6
50–59	560	19.4	1,033	35.8	354	42.6	313	37.7
60–69	826	28.6	288	10.0	150	18.0	55	6.6
70+	1,139	39.5	0	-	0	-	0	-
Median age at diagnosis (years)	66		49		49		48	
Year of diagnosis								
1961–1969	443	15.9	9	0.3	2	0.2	0	-
1970–1979	714	25.6	88	3.2	49	5.9	27	3.3
1980–1989	852	30.6	601	21.6	222	26.7	160	19.2
1990-	778	27.9	2,089	74.9	558	67.2	644	77.5
Parity								
Nulliparous/missing			421	15.1			107	12.9
1			493	17.7			124	14.9
2			1,221	43.8			381	45.9
3+			652	23.4			219	26.3
Median age at first birth (years)			25				24	

**Figure 1:**
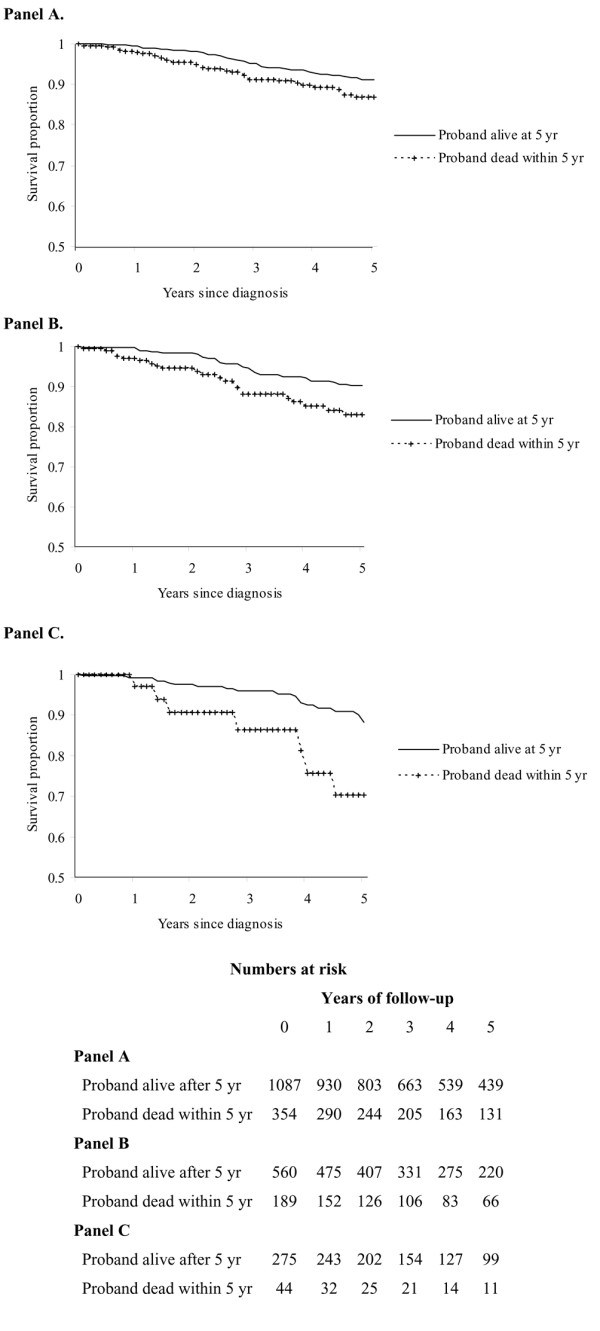
Kaplan-Meier estimates of breast cancer-specific mortality of women whose cancer was diagnosed since 1991 and who have a first-degree relative with breast cancer. Estimates are stratified by proband's cause-specific outcome. **(a) **One thousand seven hundred seventy-eight daughters with mother as proband. **(b) **Eight hundred forty-nine daughters with a mother younger than 70 years of age as proband. **(c) **Three hundred forty-eight sisters with an older sister as proband.

We categorized the proband into categories of vital status 5 years after diagnosis and studied the 5-year survival proportion in their daughters or sisters. We present Kaplan-Meier plots of patients with recently diagnosed (since 1991) breast cancer (Figure 1). The 5-year cause-specific survival proportion for daughters whose cancer was diagnosed since 1991 and whose mothers died within 5 years was 87% (95% confidence interval [CI], 82% to 91%) compared to 91% (95% CI, 89% to 93%) for daughters whose proband was alive after 5 years (log rank test, *p *= 0.03) (Figure 1a).

Mothers are older and their tumors were diagnosed in earlier calendar periods, making a comparison to sisters difficult. To compare mother-daughter pairs to sister pairs, we limited the age of the mother at diagnosis to that of the sister (that is, age of less than 70 years at diagnosis) (Figure 1b). The 5-year cause-specific survival proportion for daughters whose cancer was diagnosed since 1991 and whose mothers were younger than 70 years at diagnosis and died within 5 years was 83% (95% CI, 75% to 89%) compared to 90% (95% CI, 87% to 93%) for daughters whose proband was alive after 5 years (log rank test, *p *= 0.01). Finally, the 5-year cause-specific survival proportion for a woman whose older sister died of the disease within 5 years was 70% (95% CI, 46% to 85%) compared to 88% (95% CI, 82% to 92%) for sisters whose proband was alive after 5 years (log rank test, *p *= 0.01) (Figure 1c).

### Multivariable analyses

Using a Cox proportional hazards model, we present the 5-year breast cancer-specific mortality for daughters and sisters by proband's prognosis (good, medium, or poor) (Table 2). We estimated the 5-year cause-specific mortality of the breast cancer patients by mother or sister proband separately. We present two separate models for all pairs, one with adjustment for age at and calendar period of diagnosis, another with additional adjustment for age at first birth, parity, socioeconomic status, and area of residence. The multivariable risk to die from breast cancer in the final model was 60% higher in daughters (hazard ratio [HR] 1.6; 95% CI, 1.1 to 2.3) and 80% higher in sisters (HR 1.8; 95% CI, 1.0 to 3.4) of a proband with poor prognosis as compared to those of a proband with good prognosis. Analyzing sisters and daughters together resulted in a similarly increased risk to die from breast cancer (Table 2). To achieve comparable age range in mothers and daughters, we analyzed only pairs with mothers younger than 70 years at diagnosis. Results were similar to the unrestricted analysis (data not shown).

**Table 2:** Survival model of the cause-specific mortality of women with primary breast cancer by prognosis in proband^a^

	Deaths	Adjusted^b^	Adjusted^c^
		HR (95% CI)	HR (95% CI)
2,787 Mother-daughter pairs			
Proband prognosis^d^			
Poor (tertile 1: <33%)	84	1.6 (1.1–2.3)	1.6 (1.1–2.3)
Medium (tertile 2: 33%–66%)	83	1.4 (1.0–2.0)	1.4 (1.0–2.0)
Good (tertile 3: >67%)	53	1.0 reference	1.0 reference
Test for trend		*p *= 0.007	*p *= 0.006

831 Sister pairs			
Proband prognosis^d^			
Poor (tertile 1: <33%)	26	1.7 (0.9–3.2)	1.8 (1.0–3.4)
Medium (tertile 2: 33%–66%)	23	1.4 (0.7–2.6)	1.5 (0.8–2.9)
Good (tertile 3: >67%)	17	1.0 reference	1.0 reference
Test for trend		*p *= 0.08	*p *= 0.06

Combined (daughter and sister)			
Proband prognosis^d^			
Poor (tertile 1: <33%)	110	1.6 (1.2–2.2)	1.6 (1.2–2.2)
Medium (tertile 2: 33%–66%)	106	1.4 (1.0–1.9)	1.4 (1.1–1.9)
Good (tertile 3: >67%)	70	1.0 reference	1.0 reference
Test for trend		*p *= 0.002	*p *= 0.002

^a^Cox proportional hazards model of the 5-year cause-specific mortality of 3,618 women with primary breast cancer by prognosis in proband (mother or older sister). ^b^Adjusted for age and period of diagnosis of the daughter or younger sister. ^c^Adjusted for age and period of diagnosis, parity, age at first birth, socioeconomic status, and area of residence of the daughter or younger sister. ^d^Proband's 5-year cause-specific prognosis as defined by separated Cox proportional hazards model adjusted for proband's age and period of diagnosis. CI, confidence interval; HR, hazard ratio.

As a validation analysis, we restricted our analysis to include only all patients with breast cancer (daughters and sisters) diagnosed since 1991 (*n *= 2,590), a period with complete family links. The 5-year cause-specific mortality of the patients with breast cancer in the final fully adjusted model was 90% higher in women (HR 1.9; 95% CI, 1.3 to 2.8; *n *= 73) whose proband had poor prognosis and 70% higher (HR 1.7; 95% CI, 1.1 to 2.5; *n *= 73) if the proband had medium prognosis compared to women whose proband had good prognosis (*n *= 40; *p *for trend = 0.002). Because the findings are in agreement with the whole period analysis, the results are in support of a high internal validity.

We analyzed the importance of age at diagnosis by calculating the concordance proportion of prognosis within mother-daughter pairs (Table 3). The overall concordance in prognosis (good, medium, or poor) between mother-daughter pairs was strongest among pairs with mothers whose cancer was diagnosed at less than 40 years of age (*p *for trend <0.001). When analyzing the poor prognosis concordance, the trend by age was equally significant.

**Table 3:** Concordance in 5-year breast cancer-specific prognosis (good, medium, or poor) among 2,787 mother-daughter pairs^a^

Age of mother at diagnosis (years)	Concordance in poor prognosis		Overall concordance in prognosis	
	
	Events	Proportion	Events	Proportion
<40	12/13	0.92	37/43	0.86
40–49	60/66	0.91	166/264	0.62
50–59	129/147	0.88	337/522	0.64
60–69	211/251	0.84	502/819	0.61
70+	324/442	0.73	613/1,139	0.53
All ages	736/919	0.80	1,656/2,787	0.59
Test for trend	*p *< 0.001		*p *< 0.001	

^a^Concordance in 5-year breast cancer-specific prognosis (good, medium, or poor) by age at diagnosis in mother among mother-daughter pairs as defined by Cox proportional hazards models.

## Discussion

Our results suggest that the prognosis of breast cancer has an inherited component. The survival of women with familial breast cancer is indeed predicted by the prognosis of her first-degree relative with breast cancer. Contrasting women with relatives of really good prognosis to those of poor prognosis showed a 60% to 80% higher mortality rate among first-degree relatives of women with a relative with poor prognosis. The concordance in prognosis was more pronounced in women with first-degree family members whose cancer was diagnosed at a young age.

Strengths of our study include the large size, the population-based prospective design, the unbiased definition of family history and outcome, as well as the completeness of follow-up. A complicated issue is whether the lack of information of clinical covariates (such as stage of disease, hormone receptor status, histopathologic features, and ploidy) limits the validity of our findings. We argue that adjusting for such factors in the analysis is inappropriate. If familial clustering of prognosis reflects a genuine biologic phenomenon, it would be mirrored in established prognostic factors. Indeed, if we could measure the biologic aggressiveness of the tumor precisely, adjustment would eliminate the association completely. Still, information on clinical covariates and, in the best possible scenario, information on *BRCA1*, *BRCA2*, and *CHEK2 *(CHK2 checkpoint homolog [*Schizosaccharomyces pombe*]) might have allowed a deeper understanding of the biologic mechanisms by which prognostic outcome is determined by breast cancer survival among previously affected relatives. Confounding by suboptimal treatment within some families is a more serious concern discussed below.

Before we accept familial clustering of breast cancer prognosis as a genuine biologic phenomenon, several rival interpretations need consideration. Unavoidably, some time normally lapses between detectability – typically through self-examination – and clinical diagnosis followed by treatment of breast cancer. This delay is likely affected by factors such as awareness, denial, socioeconomic status, and access to health care. Though these factors are likely to be correlated among first-degree relatives, several circumstances argue against these factors as major contributors to our findings. Our estimates on inheritance of prognosis in the adjusted model, including socioeconomic status and area of residence, are robust (Table 2). Furthermore, self-examination has never been documented to measurably reduce breast cancer mortality [18] and Sweden has a public health care system available to everyone at minimal cost. Hence, difference in access to health care is an unlikely explanation to major differences in breast cancer prognosis between families. Finally, awareness of breast cancer might be higher among first-degree relatives of a woman who died rather than among relatives of someone who was cured of breast cancer. If anything, such awareness should produce results opposite to those we found [19,20].

Secondly, familial clustering in the use of mammography screening might contribute to the pattern of results that we found. Again, several arguments make this explanation less likely. During the late 1980s and 1990s, service screening with mammography first became widely available to all Swedish women in relevant ages. Furthermore, the mortality difference we observed between families is substantially larger than the documented benefit from regular mammography screening, notably among women younger than 50 years of age [16]. It is true that breast density, which affects mammographic sensitivity [21,22], has a strong hereditary component [23,24]. Though unlikely as a main contributor to our findings, this issue deserves further investigation.

A third conceivable confounding factor in our data is access to and compliance with optimal treatment. As already emphasized, equality of health care is widely perceived as excellent in Sweden and social gradients in breast cancer survival are much smaller than the associations we found among relatives [25]. Finally, obesity has been shown to affect breast cancer prognosis [13]. A correlation in body weight among family members may have affected our findings but would have been unlikely to substantially affect our results [20]. Another factor that has been associated with both risk and prognosis is HRT [26,27], but correlation in HRT use across generations is not expected to be substantial.

Our reasoning leads us to consider familial clustering of breast cancer prognosis as a possible biologic phenomenon rather than a consequence of confounding by factors such as awareness, screening behavior, or treatment. Theoretically, such clustering might arise as a consequence of several mechanisms. According to our main hypothesis, germline genetic variation may be relevant not only for risk of transformation and cancer incidence, but also for prognostically important features of the malignant phenotype. Whereas most studies confirm a poor prognostic outlook among *BRCA1 *mutation carriers [6,28], some studies present conflicting results with regard to a poor prognosis in women with high-penetrant mutations [7]. Additionally, little is presently known about the possible impact of low-penetrant genetic alterations that will constitute the majority of the genetic variation in familial breast cancer [29]. Hence, this area deserves further scientific inquiry.

Another, more speculative, possibility is familial differences in the ability to tolerate and/or respond to systemic treatment [30]. Although low tolerance seems less likely for hormonal manipulation (notably for treatment with antiestrogens, which has limited side effects), it might be more relevant for chemotherapy [31,32]. Because systemic chemotherapy impacts cure rates only in an adjuvant setting [31], we would expect effects to be confined to younger women in recent decades, when such treatment became standard among premenopausal patients. However, the familial component of prognosis was not confined to any age group or time period.

The inheritance of breast cancer prognosis has been studied both in animal models of mammary cancer and in genetic association studies [33,34]. Results from mouse models suggest that strain background is a significant determinant of experimental mammary carcinoma behavior [34-36]. However, the question of whether germline variation in human tumor suppressor genes or oncogenes affects breast cancer progression is still unanswered [34]. Several investigators have explored the possible relationship between breast cancer survival and genetic polymorphisms in growth factor receptors as well as genes involved in angiogenesis, xenobiotic metabolism, DNA repair, and the cell cycle checkpoints. Many studies were based on small sample size, and to our knowledge, few of the observed associations have been confirmed. One of the largest studies to date assessed the association of polymorphisms in 22 DNA repair, hormone metabolism, carcinogen metabolism, and other genes with breast cancer survival in a population-based study [37]. The authors observed the largest effect on decreased survival for a gene involved in DNA double-strand break repair, *LIG4 *(ligase IV). A recently published study has demonstrated that metastatic efficiency is modulated by the GTPase-activating protein encoded by *Sipa1 *(signal-induced proliferation-associated gene 1) [38].

## Conclusion

We conclude that information about the outcome of breast cancer among affected first-degree relatives conveys prognostic information relevant to women with newly diagnosed breast cancer. This novel observation might become relevant for clinical management provided that the post-prognostic information can be shown to be independent of that from established predictors of outcome. Further research into the likely genetic determinants of inherited breast cancer prognosis might also provide new biologic insight. Similar studies of other cancer sites should also be a high priority.

## Abbreviations

BRCA1 = breast cancer 1, early onset; CI = confidence interval; HR = hazard ratio; HRT = hormone replacement therapy.

## Competing interests

The authors declare that they have no competing interests.

## Authors' contributions

MH had full access to all of the data in the study and takes responsibility for the integrity of the data and the accuracy of the data analysis, was responsible for the study concept and design, acquired the data, analyzed and interpreted the data, drafted the manuscript, critically revised the manuscript for important intellectual content, and was responsible for the statistical analysis. PH was responsible for the study concept and design, analyzed and interpreted the data, drafted the manuscript, and critically revised the manuscript for important intellectual content. KC was responsible for the study concept and design, acquired the data, analyzed and interpreted the data, drafted the manuscript, critically revised the manuscript for important intellectual content, was responsible for the statistical analysis, and obtained funding for the study. PWD was responsible for the study concept and design, analyzed and interpreted the data, critically revised the manuscript for important intellectual content, and was responsible for the statistical analysis. LL acquired the data, analyzed and interpreted the data, critically revised the manuscript for important intellectual content, and was responsible for the statistical analysis. H-OA analyzed and interpreted the data and critically revised the manuscript for important intellectual content. All authors read and approved the final manuscript.
